# Lymph node retrieval in abdominoperineal surgical specimen is radiation time-dependent

**DOI:** 10.1186/1477-7819-4-29

**Published:** 2006-06-02

**Authors:** Alain Sermier, Pascal Gervaz, Jean F Egger, My Dao, Abdelkarim S Allal, Marta Bonet, Philippe Morel

**Affiliations:** 1 Department of Surgery, University Hospital Geneva, Switzerland; 2 Department of Pathology, University Hospital Geneva, Switzerland; 3 Department of Radiation Oncology, University Hospital Geneva, Switzerland

## Abstract

**Background:**

A low yield of lymph nodes (LN) in abdominoperineal resection (APR) specimen has been associated with preoperative radiation therapy (XRT) in population-based studies, which may preclude adequate staging of anorectal carcinomas. We hypothesized that the number of LN retrieved in APR specimen was correlated with the dose and the timing of pelvic irradiation.

**Patients and methods:**

We performed a retrospective study of 102 patients who underwent APR in a single institution between 1980 and 2004. Pathological reports were reviewed and the number of lymph nodes retrieved in APR specimens was correlated with: 1) Preoperative radiation; 2) Dose of pelvic irradiation; and 3) Time interval between the end of XRT and surgery.

**Results:**

There were 61 men and 41 women, with a median age of 66 (range 25–89) years. There were 12 patients operated for squamous cell carcinoma of the anal canal (SCCA) and 90 for rectal cancer. 83% and 46% of patients with anal and rectal cancer respectively underwent radical/neoadjuvant radiotherapy. The mean ± SD number of LN in APR specimen was 9.2 ± 5.9. The mean number of LN in APR specimen was significantly lower in patients who underwent preoperative XRT (8 ± 5.5 vs. 10.5 ± 6.1, Mann-Whitney *U *test, p = 0.02). The mean number of LN was not significantly different after XRT in patients with SCCA than in patients with rectal cancer (6.2 ± 5.3 vs. 7.8 ± 5.3, p = 0.33). Finally, there was an inverse correlation between the yield of LN and the time elapsed between XRT and surgery (linear regression coefficient r = -0.32, p = 0.03).

**Conclusion:**

Our data indicate that: 1) radiation therapy affects the yield of LN retrieval in APR specimen; 2) this impact is time-dependent. These findings have important implications with regard to anatomic-pathological staging of anal and rectal cancers and subsequent decision-making regarding adjuvant chemotherapy.

## Introduction

Adequate surgical lymphadenectomy and pathological evaluation of lymph nodes is a prerequisite for tumor staging and subsequent decision regarding adjuvant chemotherapy in patients with loco-regionally advanced rectal cancer [1]. In the 1997 TNM classification, of both the American Joint Committee on Cancer (AJCC) and the International Union Against Cancer (UICC), it is recommended that histological examination of a colorectal carcinoma (CRC) specimen should include a minimum of 12 lymph nodes [2]. This statement, however, was not intended to be a requirement for pN0, but rather a guideline, and it appears that these criteria are met only in 31% of the patients with rectal cancer [3]. This has important implications in clinical practice, since examination of nine or fewer lymph nodes is related to poor prognosis in patients with node-negative CRC [4-6].

Ionizing radiation has significant effects on the morphology of lymph nodes, including lymphocyte depletion and stroma fibrosis [7]. Indeed, data from population-based cancer registry indicate that preoperative XRT may have a negative impact on the number of lymph nodes retrieved from surgical specimen [8]. Other factors, which may affect lymph nodes yield in rectal cancer specimen, include tumor size, as well as examination by a dedicated histopathologist [9-11].

The aim of this study was to better define the impact of XRT in a population of patients who underwent abdominoperineal resection (APR) with or without previous XRT. We hypothesized; 1) that the yield of LN retrieval in APR specimen was lower in patients who had preoperative XRT; 2) that the effect of radiation on LN retrieval was dose-dependant; and 3) that the impact of preoperative XRT was time-dependent (i.e. the longer the delay between XRT and surgery, the more severe the lymph node depletion).

## Patients and methods

This is a retrospective study of consecutive series of patients, who had underwent abdominoperineal resection for a histologically proven adenocarcinoma of the rectum or squamous cell carcinoma of the anal canal at University Hospital Geneva between September 1980 and February 2004. The total number of lymph nodes identified within the APR specimen was derived from the histology report in each case. Using these reports together with the patient notes the following information was recorded for each patient: age, gender, site of tumor (anal canal or lower rectum), TNM stage, preoperative radiotherapy status (short-course, long-course or none), dose of radiation delivered to the tumor, and finally the time elapsed between the end of XRT and surgical resection.

### Radiation techniques

Patients with anal cancer were irradiated according to a previously described protocol [12]. Shortly, external beam radiation therapy (EBRT) was delivered in two sequences, with generally a median gap of 6 weeks or less between the sequences. For the first sequence external beam radiation therapy (EBRT) was used to a median dose of 39.6 Gy using mega-voltage photon beams. Boost treatment consisted of an additional dose of 20 Gy to the initial involved areas either using EBRT or brachytherapy. Patients with rectal cancer were treated preoperatively to a total dose of 45–50 Gy or a biologically equivalent dose using different RT fractionations as previously described [13]. The initial target volume included the tumor and any enlarged lymph nodes, perirectal and internal iliac lymph nodes, as well as the presacral area. Generally one posterior and two lateral Mega-voltage photon beams were used.

### Surgical technique

The standardized procedure for APR included full mobilization of the rectum with sharp dissection in the plane anterior to Waldeyer's fascia down to the level of the levator muscles, in accordance with the Total Mesorectum Excision (TME) technique. Proximal section of the sigmoid colon was performed after ligature of the superior rectal artery or the inferior mesenteric artery. The perineal dissection encompassed the perianal skin, anal sphincters before dividing laterally the levators and the anal coccygeal ligament posteriorly.

### Histological technique

Dissection of the specimen was performed according to a standard operating procedure, which did not change significantly over the course of the study. Lymph nodes were identified by direct inspection and manual palpation after close transverse slicing of the mesorectum and sigmoid mesentery. Histological technique involved careful standard dissection and direct inspection. Neither fat clearance, nor sentinel node mapping methods were used in this series of patients.

### Statistical analysis

Statistical analyses were undertaken by means of the software package Statgraph 3.0 software for Windows (Statgraph Software Inc., San Diego, CA). Quantitative data were expressed as mean ± SD, or median (range). Groups' comparisons were made using Fisher's exact test for categorical variables, and Student t-test, Mann-Whitney U test or analysis of variance (ANOVA) for continuous variables. P-values less than or equal to a two-sided alpha-level of 0.05 were considered statistically significant.

## Results

There were 61 men and 41 women, with a median age of 66 (range 25–89) years. 12 patients underwent APR for SCCA and 90 for rectal cancer. Ten (83%) SCCA patients and 42 (46%) rectal cancer patients underwent preoperative radiotherapy. The clinico-pathological characteristics of patients and tumors according to preoperative XRT status are summarized in Table 1. Six patients had no evidence of residual tumor within the surgical specimen (Stage 0). There were 30 Stage I, 20 stage II, and 46 stage III patients. Of these, 44% of patients had positive lymph nodes (N1-2) in the specimen. The mean delay between the end of XRT and surgery was 151 (range 57–231) days in SCCA patients, and 49 (4–229) days in patients with rectal cancer (Mann-Whitney *U *test, p = 0.001).

**Table 1:** Clinicopathological characteristics of patients according to preoperative radiation therapy

	**CRT**	**NO CRT**	**p**
**Age **(median, range)	61.5 (25–82)	67.5 (28–89)	0.33
**Male gender (%)**	61.5	58	0.84
**Indication for APR**			**0.001**
Anal cancer	10	2	
Rectal cancer	42	48	
**LN retrieved**	8.0 ± 5.5	10.5 ± 6.1	**0.02**
**Cases with positive LN (%)**	44.2	44	1.00
**Pathological staging**			
0	6	-	
I	11	19	
II	10	10	
III	25	21	

* Student t test, Mann-Whitney U test or Fisher's exact test, when indicated

### Yield of LN retrieval in APR specimen according to XRT status

**Figure 1:**
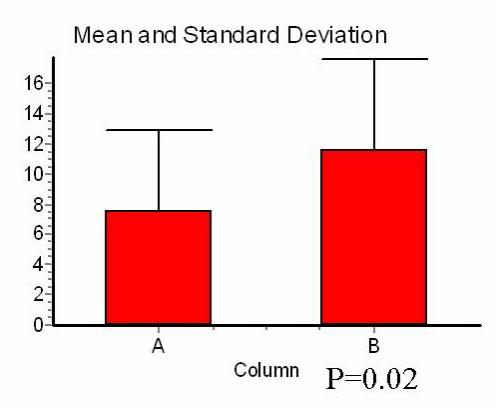
Number of lymph nodes in APR specimen according to preoperative radiation therapy status.

When considering all patients together, the mean number of LN in surgical specimen was 9.2 ± 5.9 (median 8, range 0–24). The mean number of LN in APR specimen was significantly lower in patients who underwent preoperative XRT (8 ± 5.5 vs. 10.5 ± 6.1, Mann-Whitney *U *test p = 0.02) (Figure 1).

### The effects of preoperative XRT according to the dose of irradiation

**Figure 2:**
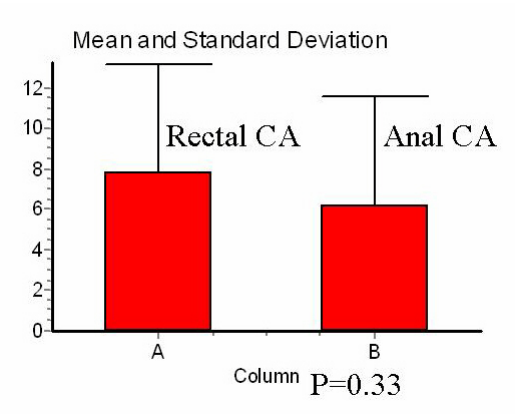
Number of lymph nodes in APR specimen according to tumor location.

We compared the number of LN in APR specimen of 10 SCCA patients (who received a mean dose to the pelvis of 60 Gy) with 42 rectal cancer patients who were treated with a mean dose of 45 Gy. The mean number of LN was lower after XRT in patients with SCCA than in patients with rectal cancer; however, this difference did not reach statistical significance (6.2 ± 5.3 vs. 7.8 ± 5.3, p = 0.33) (Figure 2).

### The effects of preoperative XRT are time-dependent

**Figure 3:**
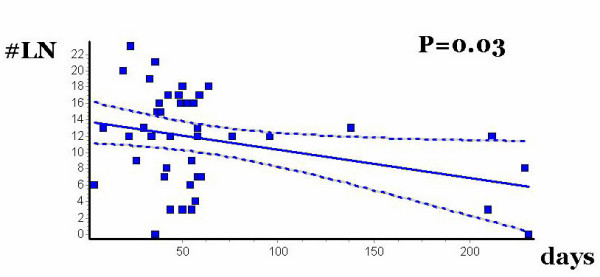
Linear regression analysis of lymph nodes in APR specimen according to interval between the end of pelvic irradiation and the time of surgery.

Finally, we performed a regression analysis of the number of LN according to the delay between the end of XRT and the time of surgery. The median time elapsed was 50 days (range 4–231), and this delay was significantly longer in patients with SCCA than in patients with rectal cancer (151 ± 79 vs. 49 ± 35 days, p < 0.001). There was an inverse correlation between the yield of LN and the time elapsed between XRT and surgery (linear regression coefficient r = -0.32, p = 0.03) (Figure 3).

## Discussion

In this series the number of LN retrieved in APR specimen of rectal cancer patients with (8 LN) or without (10.5 LN) preoperative XRT are quite similar to large population-based series from the US [3], and Europe [14], suggesting that the techniques of surgical lymphadenectomy and pathological examination used in our institution are adequate. Although this 25% reduction in LN yield may seem of little clinical significance, it is noteworthy that 31% of patients in the irradiated group had 4 or less nodes in their surgical specimen, suggesting inadequate tumor staging in one third of patients who underwent preoperative radiation therapy. Thus, our first hypothesis proved to be correct; preoperative XRT significantly and negatively affects the yield of LN in APR specimen.

By contrast, we were unable to demonstrate that our second hypothesis was correct; although patients with SCCA were irradiated with higher doses, in comparison with rectal cancer patients, the difference in the number of LN according to the tumor type failed to achieve statistical significance. Actually, (and this demonstrates our third hypothesis to be right) our data emphasizes the impact of timing, more than the actual dose of radiation, on the number of lymph nodes retrieved from APR specimen. The impact of XRT on mesorectum lymph node retrieval is more pronounced when APR is delayed by several months after the end of pelvic irradiation [15]. The regression analysis in our study was greatly influenced by the 5 patients who underwent APR for recurrent anal cancer more than 5 months after the end of radiation therapy. Consequently, current protocols with longer intervals (6–8 weeks) between XRT and surgery for rectal cancer may result in increased tumor downstaging, but also in an inadequate yield of lymph nodes in the surgical specimen [16,17].

The implications of our results are that, due to the widespread use of neoadjuvant XRT, the current TNM classification of anorectal cancers needs to be improved [18]. Pre-treatment staging using either magnetic resonance imaging or endorectal ultrasound (EUS) are accurate enough for the T stage, but have proven insufficient to determine lymph node involvement [19,20]. By comparison, and despite tumor down staging, pathological postoperative staging remained a very strong prognostic factor for both T and N stages [21,22]. Thus, the TNM staging system for rectal cancer, which currently does not take into account neoadjuvant XRT, remains pivotal for selection of patients who may benefit from additional chemotherapy, but the pathological N staging of irradiated specimen should be interpreted with caution.

## Summary

The longer is the delay between XRT and surgery, the lower is the yield of LN in the mesorectum. Future efforts for improving the yield of mesorectal lymph node after pelvic irradiation should consider reinvestigating the role of extended but selective lymphadenectomy, perhaps using modern techniques of lymph node clearing [23]. Sentinel lymph node mapping is another promising option, which needs to be assessed in prospective studies [24,25].

## Competing interests

The author(s) declare that they have no competing interests.

## Authors' contributions

AS conceived of the study; PG participated in its design and coordination and helped to draft the manuscript; JFE participated in the design of the study; MD performed the statistical analysis; ASA participated in the design of the study; MB participated in the design of the study; PM helped to draft the manuscript.

All authors read and approved the final manuscript.
